# The in vitro behaviour of canine osteoblasts derived from different bone types

**DOI:** 10.1186/s12917-019-1857-1

**Published:** 2019-04-11

**Authors:** Richard L. Meeson, Inês P. Perpétuo, Kevin Parsons, Isabel R. Orriss, Mittal Shah, Andrew A. Pitsillides, Michael Doube

**Affiliations:** 1 Clinical Services and Sciences, The Royal Veterinary College, Hawkshead Lane, North Mymms, Hatfield, Hertfordshire AL9 7TA UK; 2 Comparative Biomedical Sciences, Skeletal Biology Group, The Royal Veterinary College, Royal College Street, London, NW1 0TU UK; 3 Langford Veterinary Services, University of Bristol, Langford House, Langford, BS40 5DU UK; 4 Jockey Club College of Veterinary Medicine and Life Sciences, City University of Hong Kong, Tat Chee Avenue, Kowloon, Hong Kong; 5 Queen Mother Hospital for Animals, Royal Veterinary College, Hawkshead Lane, North Mymms, Hatfield, AL9 7TA UK

**Keywords:** Osteoblast, Femur, Trabecular, Subchondral, Cortical, Canine

## Abstract

**Background:**

Our understanding of the biology of osteoblasts is important as they underpin bone remodelling, fracture healing and processes such as osseointegration. Osteoblasts isolated from human humeral samples display distinctive biological activity in vitro, which relates to the samples’ bone types (subchondral (S), trabecular (T), cortical (C)). Our aim was to isolate primary osteoblast cultures from different bone types from the proximal femur of a clinical population of dogs presented for total hip replacement and compare the behaviour of the osteoblasts derived from different bone types, to identify a preferred bone type for isolation.

**Results:**

No differences were found for osteoblast doubling time (median for S = 2.9, T = 3.1 and C = 2.71 days, respectively; *p* = 0.33), final cell number (median for S = 54,849, T = 49,733, C = 61,390 cells/cm^2^; *p* = 0.34) or basal tissue non-specific alkaline phosphatase (TNAP) activity (median for S = 0.02, T = 0.02, C = 0.03 U/min/mg protein; *p* = 0.81) between bone types after 6 days of culture in basal media. There were no differences in mineralizing TNAP activity (S = 0.02, T = 0.02, C = 0.03 U/min/mg protein, *p* = 0.84) or in mineralized area (S = 0.05, T = 0.04, C = 0.04%, *p* = 0.92) among cells from different bone types.

**Conclusions:**

There is no significant difference in mean doubling time, basal or mineralizing TNAP activity or mineralized area in osteoblasts derived from subchondral, cortical, or trabecular bone types from the canine femoral head. However, there appears to be a high level of inter-animal variability in the studied parameters, which was independent of age, body mass, and sex. Trabecular isolate osteoblasts have the least variation of the bone types studied, and therefore should be considered a preferred source for primary osteoblast cultures. The work here provides baselines for canine osteoblast function, which has utility for future comparative studies.

## Background

Isolation of osteoblasts from adult human bone was first undertaken in the 1970s [1]. A vast amount of in vitro work has followed since and osteoblastic cell lines have been used as models for understanding numerous characteristics such as differentiation, cytokine and hormone regulation, bone matrix synthesis and the molecular mechanism of bone disease. More recently, osteoblast cultures have been important in our understanding of drug mechanisms, tissue engineering and the osseointegration of bone with implants [2]. Osteoblast cultures are also important for developing our understanding of complex processes occurring in osteoarthritis [3].

Although bone is highly conserved in terms of its development, remodelling and healing, it is clear that different regional bone types (trabecular, cortical, and subchondral) respond differently to in vivo challenges [4–6]. Variations in osteogenic potential have been demonstrated between marrow and periosteal osteoblasts from the same individual [7]. Such differences in bone type are however, often overlooked yet they remain important to consider. For example, trabecular bone, which is mainly found within the metaphyseal regions and extremities of long bones, is architecturally distinct from cortical and subchondral bone and usually provides load-bearing support with a large area over which bone (re)modelling occurs, potentially lending itself to higher metabolic remodelling than cortical bone [8, 9]. There is therefore, a need to consider whether the bone type that osteoblasts are isolated from could influence their in vitro characteristics. Work on human osteoblasts isolated from cortical, subchondral and trabecular bone types have shown significant variation in biological activity in vitro, and this effect was conserved across patients with osteoarthritis or osteoporosis [10]. Briefly, osteoblasts from subchondral and cortical bone types proliferated more rapidly than those from trabecular bone, and tissue non-specific alkaline phosphatase (TNAP) activity was enriched in trabecular human osteoblasts. Could this phenomenon be conserved across different joints and species? If so, this may have implications for in vitro primary osteoblast culture studies, in addition to clinical considerations such as osseointegration of uncemented hip replacements.

Canine bone biology is close to that of humans in terms of structure and remodelling characteristics [11–13]. There is also a developing understanding and appreciation of dogs with spontaneous disease such as osteosarcoma [14] and osteoarthritis [15]. The canine patient, with its spontaneous development of osteoarthritis, is arguably a more relevant model of human hip osteoarthritis [16–18] than induced rodent models. Unlike rodents, dogs are increasingly receiving hip replacements for end-stage joint disease. To date there have been no studies evaluating clinical samples of canine osteoblasts taken from different bone tissue types, which may have implications for implant research and development.

This study aimed to evaluate whether bone type-specific differences in osteoblast growth and differentiation activity exist within the canine proximal femur, and if so, to determine which bone type may be most appropriate for future in vitro osteoblast studies using canine samples. Primary human osteoblasts display distinct in vitro behaviours that relate to the bone type from which they were isolated, so we hypothesised that there would be significant differences in canine osteoblast behaviour between different bone types.

## Results

### Canine samples

Samples were collected as clinical residue from 11 consecutive dogs undergoing elective total hip replacement due to osteoarthritis. Seven males and four females were enrolled, with a mean age of 6.1 ± 2.9 years and mean weight of 30 ± 12 kg. Characteristics of each individual sample are listed in Table 1. Breeds included: two German shepherds, Labrador, Briard, Labradoodle, Italian Spinone, West Highland Terrier, Golden Retriever, Retriever crossbreed, and two Border Collies. These are all medium or large breeds and the only chondrodystrophic breed (i.e. with inherited breed-associated cartilage malformations) was the West Highland terrier. All dogs were returned to their owners following recovery from surgery; their clinical progress was not followed as part of this study.

**Table 1:** Sample characteristics

Canine sample	Age (years)	Sex	Body mass (kg)	Breed	Medication
Dog 1	5.6	Male	39	German shepherd	None
Dog 2	8.8	Female	33	Labrador	NSAID
Dog 3	1.0	Female	30	Briard	NSAID
Dog 4	7.0	Female	32	Labradoodle	NSAID
Dog 5	6.9	Male	47	Italian Spinone	NSAID
Dog 6	9.4	Male	10	West Highland Terrier	None
Dog 7	4.3	Male	28	Golden retriever	NSAID
Dog 8	2.3	Male	40	German shepherd	NSAID
Dog 9	9.3	Male	42	Retriever crossbred	NSAID
Dog 10	8.7	Female	13	Border collie	NSAID
Dog 11	3.8	Male	19	Border collie	NSAID

*NSAID* non steroidal anti-inflammatory drug

### Canine bone cells from different bone types have no differences in proliferation rates or basal TNAP activity

**Fig. 1:**
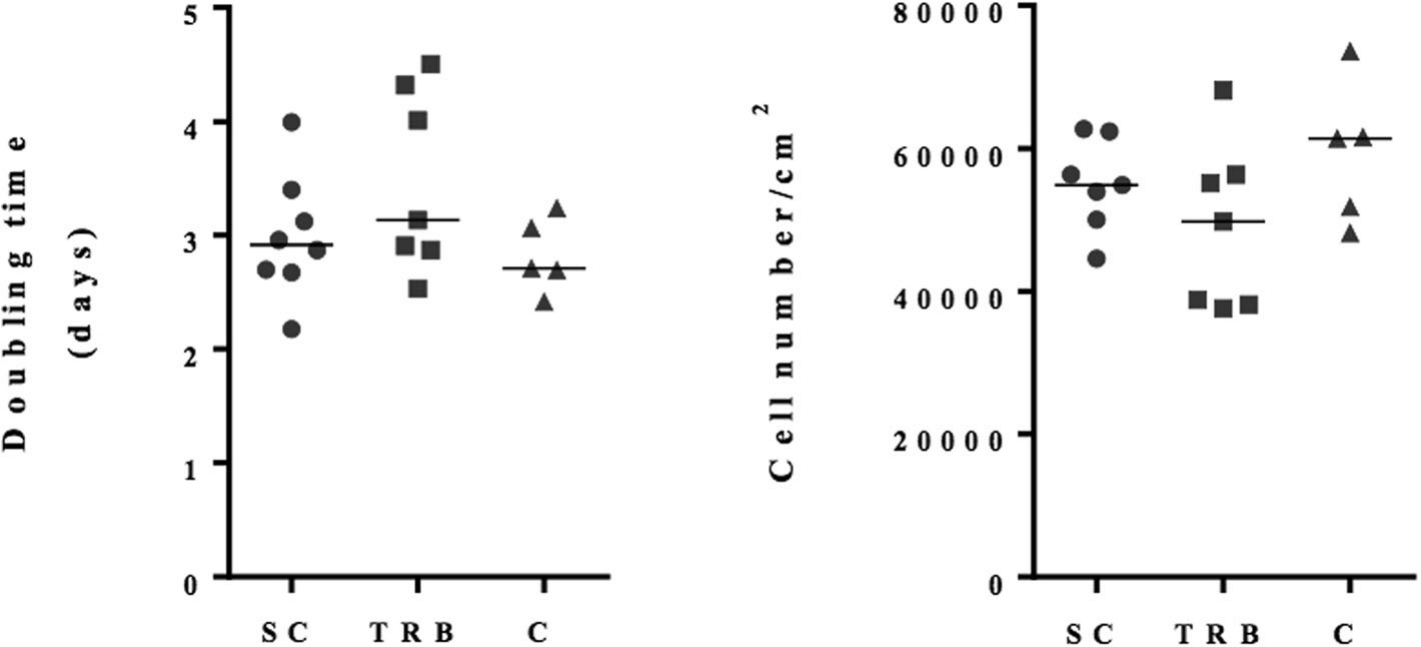
After 6 days in basal media primary osteoblasts isolated from different bone types showed wide variation but no statistically significant differences in median doubling time or final cell number. SC – subchondral, TRB – trabecular, C – cortical

**Fig. 2:**
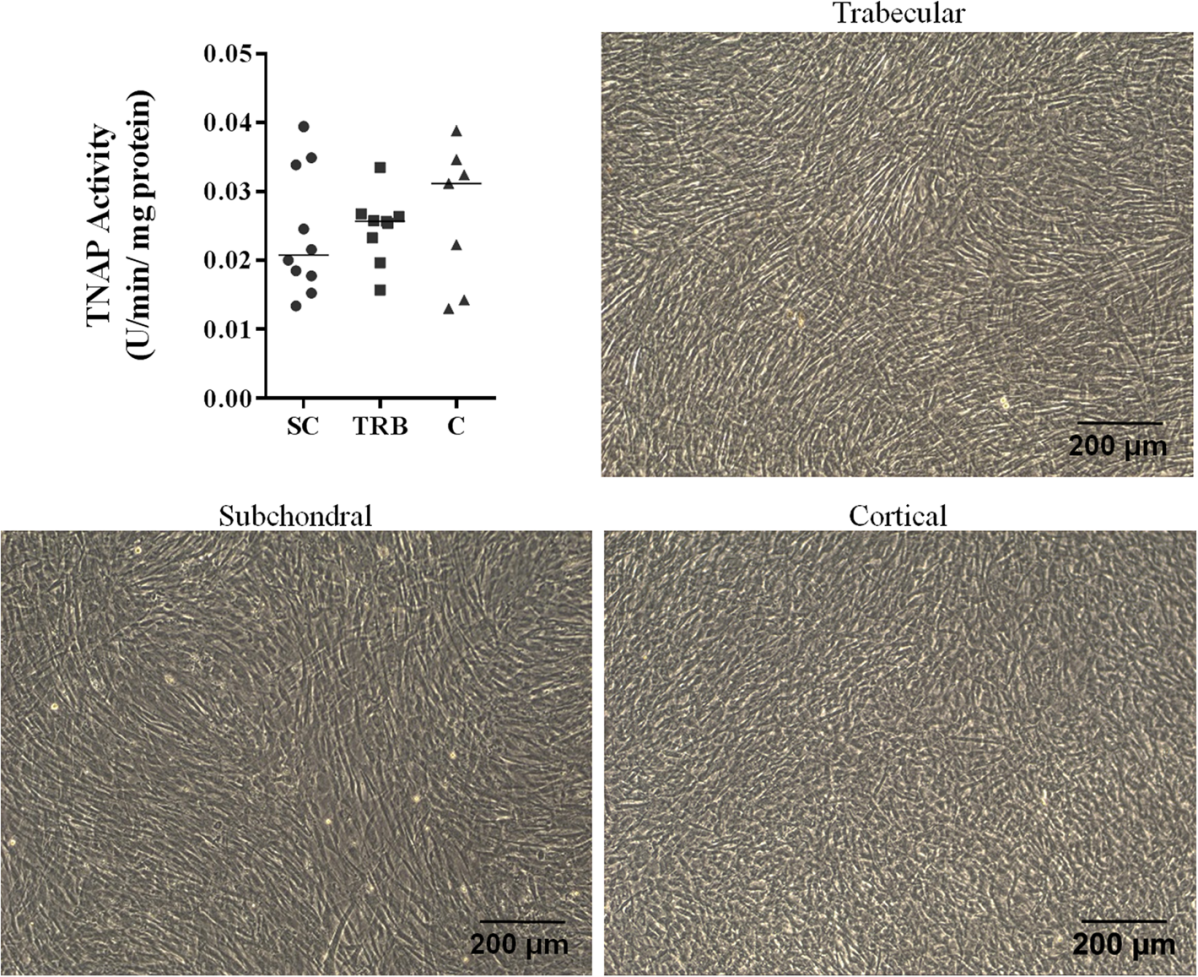
After 24 h of confluence there were no statistically significant differences in median basal TNAP activity between cells from different bone types. No morphological differences were observed in cultured cells (magnification 10x). SC – subchondral, TRB – trabecular, C - cortical

Canine osteoblasts derived from different bone types did not show significant differences in proliferation rates or basal TNAP activity. There were no statistical differences in doubling time or final cell number between osteoblasts from subchondral, trabecular or cortical bone types after 6 days of culture in basal media (Fig. 1, *p* = 0.33 and *p* = 0.34, respectively). After 24 h of confluence, cells were assessed for basal TNAP activity and there was no difference in enzymatic activity between osteoblasts from subchondral, trabecular or cortical bone types (Fig. 2 *p* = 0.81).

### Canine bone cells from different bone types have no differences in mineralizing ability or TNAP activity at mineralization

**Fig. 3:**
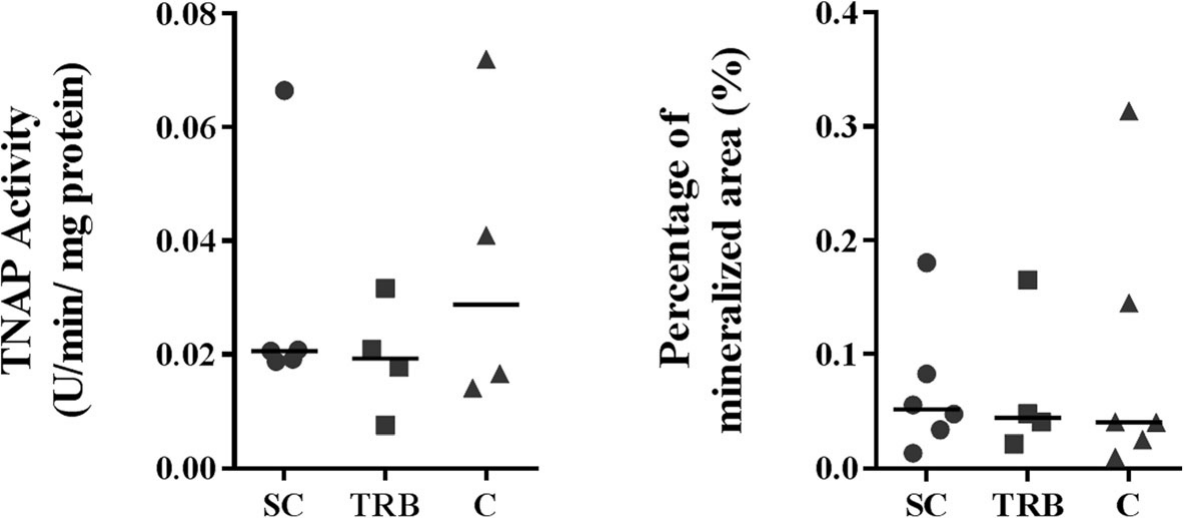
After 40 days in mineralising media primary osteoblasts isolated from different bone types showed no differences in TNAP activity or in the percentage of mineralized area. SC – subchondral, TRB – trabecular, C – cortical

We analysed both the mineralized area and the TNAP activity of the canine osteoblasts after maintenance for 40 days in mineralizing media. We found no differences in either TNAP activity (*p* = 0.84) or in the mineralized area (*p* = 0.92, Fig. 3).

### Individual dog characteristics do not explain variance in the studied parameters

Individual dog characteristics (age, gender, body mass or bone type) did not explain cell number, variance in basal or mineralizing TNAP activity or in mineralized area. To understand if the osteoblasts from subchondral, trabecular or cortical bone types exhibited cellular characteristics that were influenced by dog size, age, gender or bone type, a linear mixed model analysis was used to identify the main effects and the results are summarized in Table 2. None of the individual dog characteristics or bone type significantly explained the variance observed in cell number, mineralized area or basal and mineralizing TNAP activity.

**Table 2:** Individual canine characteristics do not explain variation in cell number, TNAP activity or nodule formation. Fixed effects were used in all cases. *p*-values are shown

Variable	Cell number	Basal TNAP activity	TNAP activity at mineralization	Mineralized area
Sex	0.445	0.271	0.613	0.116
Age (years)	0.340	0.391	0.787	0.471
Body mass (kg)	0.266	0.166	0.292	0.536
Bone site	0.116	0.448	0.717	0.877

## Methods

### Patients and sample preparation

Client-owned clinical veterinary patients undergoing treatment for spontaneous hip osteoarthritis, diagnosed radiographically, and subsequently selected for total hip replacement based on clinical and radiographic evaluation were prospectively enrolled. Only skeletally mature dogs that were greater than 10 kg in body mass (range 10–47 kg), with no known concurrent medical conditions were included. Patient data that was recorded included: age, weight, breed and concurrent medication either related or unrelated to the hip osteoarthritis. All dogs received perioperative intravenous cephalexin, 60–120 min prior to sample acquisition. Aseptic surgical technique was employed and a femoral head with proximal neck ostectomy was performed by means of an oscillating sagittal saw (Compact Air Drive II System, DePuy Synthes). The resected tissue was aseptically transferred from the operating theatre in sterile phosphate-buffered saline (PBS) with 10% antibiotic-antimycotic (AB/AM - penicillin, streptomycin, amphotericin B; Invitrogen, Paisley, UK). Samples were then kept at 4 °C for up to 24 h prior to processing. Informed consent was obtained from the dogs’ owners by means of a residual tissue clause in the client consent form with appropriate institutional ethical review board approval (URN 20181829–2).

### Preparation of explants and subchondral, trabecular and cortical bone primary osteoblasts

The excised femoral head was cut in the frontal plane with an IsoMet® Low Speed Diamond Saw (Buehler, Coventry, UK) to facilitate access to subchondral, trabecular, and cortical bone types. Subchondral bone was prepared by removing the overlying cartilage with a scalpel and then isolated using rongeurs. Trabecular bone was collected from the epiphysis avoiding the growth plate area. Cortical bone was collected from the femoral neck region. The pieces of bone from each type were cut into 1 mm^3^ explants and vortexed separately, at least six times in PBS (Gibco, Paisley, UK) containing 10% AB/AM to remove bone marrow, blood and other debris. The following isolation procedure was previously described for human humeral samples [10, 19] and applied with some modifications. Briefly, the bone pieces were incubated with 1% trypsin (Gibco, Paisley, UK) for 10 min at 37 °C. The trypsin was discarded and fragments were then washed in Dulbecco’s modified Eagle’s medium (DMEM) (Gibco, Paisley, UK) and in PBS. Bone pieces were incubated in 0.2% collagenase type II (Sigma-Aldrich, Dorset, UK) for 30 min at 37 °C; the supernatant was discarded and fragments were washed in PBS and then in DMEM (low glucose, pyruvate, no glutamine, no phenol red). The fragments were then seeded in 75 cm^2^ culture flasks (Nunc, UK) in DMEM supplemented with 10% fetal calf serum (Invitrogen, Paisley, UK), 2 mM L-glutamine (Invitrogen, Paisley, UK), 100 U/mL AB/AM (Invitrogen, Paisley, UK) as a basal media at 37 °C, 5% CO_2_. Cultures were left for 7 days without media change and thereafter, a half media change was performed every 4 days until confluent. At confluence, cells were trypsin-digested (Invitrogen, Paisley, UK) and sub-seeded in six replicates in basal media in 12 or 6-well plates (Nunc, UK) at 50000 cells/well, and the media was changed twice a week. Cells from the different bone types were seeded as described above and maintained in basal media until confluence. At 24 h post-confluence cells were changed into mineralizing media which consists of basal media plus 2 mM β-glycerophosphate (Sigma-Aldrich, Dorset, UK) and 200 μg/mL of ascorbate (Sigma-Aldrich, Dorset, UK). Media was changed twice a week until day 40 after confluence.

### Cell number and viability

Crystal violet binding to DNA was used to calculate the number of viable cells in culture, based on the principle that dead cells are no longer attached to the cell culture plastic. Briefly, cells from the different bone types were seeded into 12-well plates at 50000 cells/well, with six replicates per region, per dog sample. Cells were maintained in basal media for 6 days before assessing cell number. At day six, cells were washed with PBS and fixed with 4% paraformaldehyde (Sigma-Aldrich, Dorset, UK). Cells were stained with 0.1% crystal violet (Sigma-Aldrich, Dorset, UK) in distilled water for 30 min at room temperature. Cells were subsequently washed with distilled water to remove excess dye and the bound stain was eluted from cells with 1 mL of 10% acetic acid (Sigma-Aldrich, Dorset, UK). The quantity of nuclear stain was assessed by transferring 200 μL of the acetic acid solution to a 96 flat well plate (Nunc, UK) and the absorbance was measured at 595 nm in a Tecan M200 Pro Plate Reader (Tecan Trading AG, Switzerland). A standard curve was prepared with known serial dilutions of canine cells that were stained. Absorbance was used as a direct measure of cell nucleus staining, and cell number was extrapolated from the standard curve.

### Tissue non-specific alkaline phosphatase (TNAP) activity assay

Cell lysates were collected 24 h after confluence or, at the same time, plates were fixed for analysis from each individual well. In distilled water, cells were scraped off the plate and the cell suspension was centrifuged. A modified version of the SensoLyte® pNPP Alkaline Phosphatase Assay Kit (AnaSpec, Belgium) was used, where 50 μL of cell lysate supernatant was incubated with the pNPP chromogenic liquid substrate and absorbance was measured at 37 °C, 405 nm at regular intervals in a Tecan M200 Pro Plate Reader (Tecan Trading AG, Switzerland). The cell lysate was also used to measure protein content with the Bradford reagent (Sigma-Aldrich, Dorset, UK) according to the manufacturer’s instructions.

### Mineralization analysis

Cells which had been maintained in mineralizing media in 6-well plates for 40 days were fixed and analysed using the methods described previously [19]. Briefly, plates were washed in PBS, fixed for 5 min at room temperature with 2.5% Gluteraldeheyde (Sigma-Aldrich, Dorset, UK) and then washed again with PBS and with 70% ethanol. When dry, plates were scanned at 2400 dots per inch (dpi) on a high-resolution flat-bed scanner for analysis of mineralized nodule formation. ImageJ (http://rsbweb.nih.gov/ij/) was used to determine the surface area of mineralized bone nodules. Images were binarized and subjected to automated analysis, using constant threshold and minimum particle levels.

### Statistical analysis

For each assay, six replicates of each culture were pooled and the mean used for statistical purposes. D’Agostino-Pearson tests were used to determine normality of distributions. Kruskall-Wallis and Dunn’s post-hoc tests were used to compare samples. Linear and non-linear regressions were used to calculate correlations between cell number and TNAP activity and to determine the contribution of other sample characteristics such as age or weight of dog. A linear mixed model was used to identify the relationship between sex, age, weight and bone type (fixed factors) and the studied parameters (cell number, basal and mineralizing TNAP activity and mineralized area). The model residuals were assessed for normality. Fixed effects were evaluated individually first before combining them in a multivariable model. Statistical analysis was performed using GraphPad Prism 7.01 for Windows (GraphPad Software, La Jolla, CA, USA) and IBM-SPSS version 22 (IBM, USA). *P*-values under 0.05 were considered significant. We assumed a TNAP activity effect level from previous work [10] and calculated that six samples would be required in order to achieve a power of 80% at the 5% significance level.

## Discussion

Isolated canine osteoblasts, from all bone types showed a wide variation in activity between individual dogs. This is the first time that clinical patients have been the source of bone tissue for osteoblast isolation, and may indicate that although there are highly desirable aspects in looking towards spontaneous disease models to develop our understanding, there is increased ‘noise’ or variation present than in experimental models. Focussing on a single breed or group of dogs may also reduce the variation seen but could reduce the relevance to the dog population at large. Nonetheless, osteoblast-like cells were successfully isolated and trabecular-sourced osteoblasts had the least variation between individuals and may be a preferred osteoblasts source for future studies.

Cells isolated by explant outgrowth will be heterogeneous; early osteoblasts will likely predominate, with accelerated maturation in response to the supplemented factors, such as glucocorticoids, vitamin D, or other growth factors. The application of identical cell isolation protocols to different samples can yield osteoblast cultures that vary in terms of whether early or mature osteoblasts predominate [10, 19, 20]. It is possible that despite using identical cell isolation methods here as used previously, that the variety of breeds, ages and body weights led to divergence in specific osteoblast populations derived from each bone type in each dog. We detected substantial inter-animal variation in the studied parameters (doubling time, TNAP activity - basal or mineralizing, and mineralization), however, the variation was not directly related to dog size, age or sex. We observed smaller differences in parameter means than found previously in human humeri [10] and therefore the size of our cohort may not have been large enough to detect a smaller effect size within the greater noise of individual variation. To observe a 20% effect size based on power 0.8 and significance of 0.05, in our studied parameters within the variation we measured, we would have to increase our cohort to 100 dogs. Another option to minimise the background differences between bone sites would be to use larger groups of animals (6–10) from the same breed. Our findings suggest that there is greater diversity among dogs’ osteoblast behavior than among human osteoblast behaviour, potentially exacerbated by breed. Therefore, care needs to be taken when making generalizations based on canine osteoblast explants from clinical cohorts with spontaneous orthopaedic disease. Whether or how the presence of hip osteoarthritis influenced osteoblast behaviour diversity also remains unclear, however, all samples were osteoarthritic. Although non-diseased samples may have shown site-specific differences in activity, non-diseased hip tissue is difficult to obtain. The options include experimental animals, non-repairable hip fractures treated with ostectomy or cadaveric samples. It was not the intention of this *one medicine* orientated study to use experimental animals, and hip fractures/luxations are best primarily repaired where possible and hence samples are rare. Cadaveric samples are also problematic for cell viability and infection. Importantly, the differences seen in the human study [10] were shown in diseased samples and hence diseased canine samples were felt to be appropriate. It is important to note however, that this paper cannot make direct comparisons with the human study in terms of cellular activity (TNAP, cellular activity) as the cells were isolated from different anatomical sites and used similar but not identical cell culture methods.

In contrast to human bone type osteoblasts isolated from the humeral head, osteoblasts derived from the canine proximal femur exhibit a conserved, programmed difference in bone type-related cell in vitro behaviour. A lack of conservation of bone type-related behaviour from the canine femur to the human humerus could potentially be explained by one of a range of mechanisms such as effect of load, variations in bone turnover between bone sites (humerus vs femur), different species and effect of disease on cell culture characteristics. The location from which bone is sampled and its individual loading environment may explain the lack of pre-programmed bone type-related behaviour seen in the canine femur compared with the human humeral head [10]. Mechanical load can drive bone adaptation in structure, through the proposed actions of mechano-sensitive osteocytes that produce signalling molecules, such as NO, which in turn affect recruitment, activity and differentiation of osteoclasts and osteoblasts [21–26]. The human osteoblasts against which we compare our results were isolated from the humeral head. Due to humans’ bipedal gait osteoblasts in the humeral head are subject to net muscular forces without additional ground reaction forces, unlike the weight-bearing hip joint [9, 27]. Studies have shown that shoulder joint loading is 50–90% of body weight during abduction [28, 29], whereas the canine hip is at least one times body weight [30, 31]. Therefore, a reduced loading scenario in the shoulder as compared with the hip joint could influence the anabolic response of bone cells to those differing loads and hence the bone-type related behavioural pattern may be abolished in the canine hip samples. Further studies should address bone type-specific osteoblast activity in the human proximal femur.

This study used clinical veterinary patients with spontaneous hip osteoarthritis. Species differences in osteoblast biology are well established, so it remains to be established whether these current results could necessarily be extrapolated to humans [2, 19]. Currently, there is little information available as to how closely the veterinary clinical disease resembles human hip osteoarthritis, however, in terms of size, lifestyle, longevity, disease development, and treatment options, the dog is certainly closer to human than the rodent. In terms of bone structure, the dog remains the closest non-primate animal species to humans, however, clear differences in the rate at which bone remodelling occurs, with dogs having a significantly higher turnover rate than humans [32]. Additionally, quantitative histology of trabecular bone has shown variable rates of turnover between different locations such as lumbar compared with talar trabecular compartments, being 200 and 12% per annum, respectively. A further reason for selecting the canine femur was the biology of osteoblast type in this location could have translational impact on osseointegration of hip replacement prostheses, and a clinical source of these naturally osteoarthritic samples was available. Future work to determine the influence of bone-type on osteoblast behavior would require further comparison between samples derived from the human femoral head and or canine humeral head.

## Conclusion

There is a large individual variability between dogs in inherent biological activity of osteoblasts, independent of their type; trabecular, cortical and subchondral. However, the trabecular bone type osteoblasts had the least variation and should be considered the preferred source of osteoblasts for in vitro studies. The work in this study also provides a methodology for research groups looking to isolate and expand canine osteoblasts, as well as providing baseline data for future explant studies from canine donors. This is important in context of ‘one medicine’, whereby large animals with spontaneous natural disease are increasingly proposed as a missing link between rodent research and human clinical translation.

## Abbreviations

AB/AM: Antibiotic-antimycotic
C: Cortical
DMEM: Dulbecco’s modified Eagle’s medium
NSAID: Non-steroidal anti-inflammatory drug
PBS: Phosphate-buffered saline
SC: Subchondral
TNAP: Tissue non-specific alkaline phosphatase activity
TRB: Trabecular
